# Radiologically Evident Oesophageal Dilation in Paediatric Rumination Syndrome With Recurrent Aspiration Pneumonia: A Rare Case Without Achalasia

**DOI:** 10.1002/rcr2.70492

**Published:** 2026-02-02

**Authors:** Akina Nigi, Keisuke Iwamoto, Hidetoshi Itani, Shigeto Kondou

**Affiliations:** ^1^ Department of Respiratory Medicine Japanese Red Cross Ise Hospital Ise Mie Japan

**Keywords:** achalasia, aspiration pneumonia, oesophageal dilation, rumination syndrome

## Abstract

A 15‐year‐old autistic boy with rumination syndrome presented with progressive oesophageal dilation on CT. Achalasia was excluded based on imaging. This case illustrates a rare example of structural change likely due to behavioural disorder and highlights the diagnostic value of CT when manometry is unfeasible.

## Graphical abstract

We present a rare example of structural change likely due to behavioural disorder in a 15‐year‐old autistic boy with rumination syndrome who presented with progressive oesophageal dilation on CT. Achalasia was excluded based on imaging.

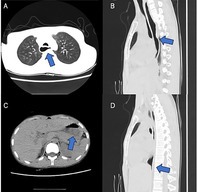

A 15‐year‐old boy with severe intellectual disability and autism had long‐standing rumination behaviour with recurrent vomiting and aspiration pneumonia. Chest CT revealed progressive, air‐filled oesophageal dilation without food stasis, while gastric contents were preserved (Figure 1A–D). Endoscopic or manometric evaluation could not be performed because of his severe autistic condition, but the absence of food residue, fluid level, or distal tapering suggested no overt achalasia or obstruction. Repetitive rumination behaviour, involving abdominal straining and air swallowing, may transiently increase intraesophageal pressure, resulting in functional dilation. While rumination syndrome is classically defined as a behavioural disorder without structural abnormality, secondary changes such as reflux esophagitis or mild dilation have been reported [1, 2]. This case highlights that severe and persistent rumination is likely to have induced functional oesophageal dilation, suggesting that behavioural mechanisms may potentially cause morphological changes even without primary motility disorder. To our knowledge, there have been no previous reports of radiologically evident oesophageal dilation in rumination syndrome without evidence of achalasia or obstruction. Although this may appear theoretically plausible due to chronic aerophagia and straining, such structural consequences of behavioural disorders remain rarely documented.

**FIGURE 1:**
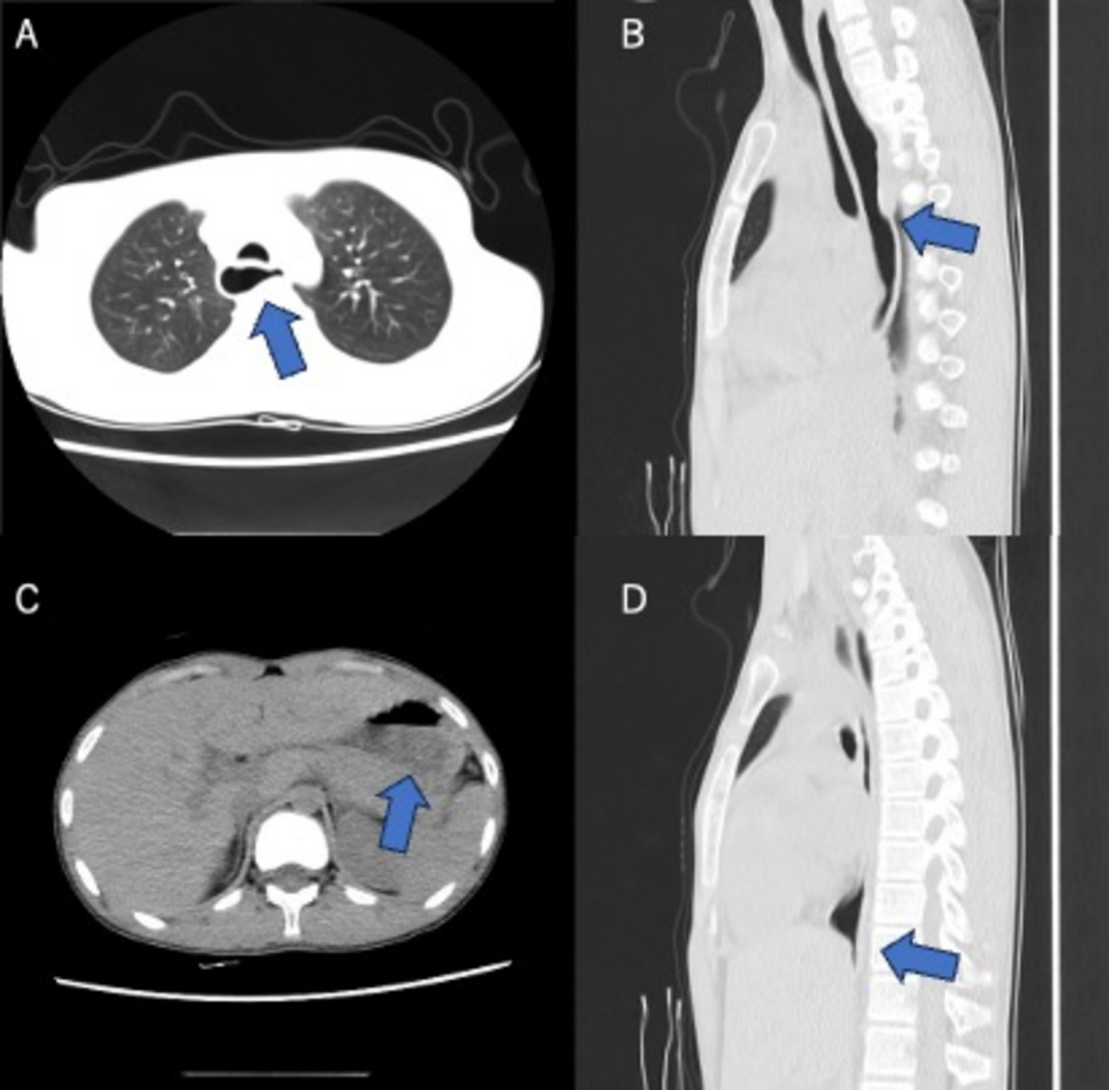
(A–D) CT imaging demonstrates persistent oesophageal dilation filled with air, without food retention or distal tapering, in a patient with rumination syndrome. (A) Axial view of upper oesophagus. (B) Sagittal view shows dilation along the thoracic spine. (C) Food residue that has passed through the oesophagus is observed in the stomach. (D) Sagittal image showing open gastroesophageal junction with no tapering.

## Consent

The authors declare that written informed consent was obtained for the publication of this manuscript and accompanying images using the consent form provided by the Journal.

## Conflicts of Interest

The authors declare no conflicts of interest.

## Data Availability

Data sharing not applicable to this article as no datasets were generated or analysed during the current study.
